# High-intensity interval training may reduce depressive symptoms in individuals with schizophrenia, putatively through improved VO_2_max: A randomized controlled trial

**DOI:** 10.3389/fpsyt.2022.921689

**Published:** 2022-08-04

**Authors:** Gry Bang-Kittilsen, John Abel Engh, René Holst, Tom Langerud Holmen, Therese Torgersen Bigseth, Eivind Andersen, Jon Mordal, Jens Egeland

**Affiliations:** ^1^Division of Mental Health and Addiction, Vestfold Hospital Trust, Tønsberg, Norway; ^2^Oslo Centre for Biostatistics and Epidemiology, University of Oslo, Oslo, Norway; ^3^Faculty of Humanities, Sports and Educational Science, University of Southeast Norway, Horten, Norway; ^4^Department of Psychology, University of Oslo, Oslo, Norway

**Keywords:** schizophrenia, treatment, exercise, cardiorespiratory fitness, high-intensity interval training, active video gaming, symptoms, depressive symptoms

## Abstract

**Introduction:**

High-intensity interval training (HIIT) may improve cardiorespiratory fitness (CRF) and mental health. The current observer-blinded RCT investigates the sparsely studied efficiency of HIIT in reducing psychotic and non-psychotic symptoms in schizophrenia and complements previous studies by investigating whether symptom reduction following HIIT is associated with, putatively partly mediated by, increased VO_2_max.

**Methods:**

Participants (outpatients meeting diagnostic criteria for schizophrenia) were randomized to HIIT (*n* = 43) or a comparison group performing low-intensity active video gaming (AVG) to control for social interaction (*n* = 39). Both interventions consisted of two supervised sessions/week for 12 weeks and a 4 months follow-up. Effects on overall symptoms and symptom domains [PANSS (0–6 scale), five-factor model] were estimated using mixed-effects models (intention-to-treat, *n* = 82). Underlying mechanisms were analyzed using moderated mediation analyses (*n* = 66). We anticipated that HIIT would reduce overall symptoms, particularly depressive symptoms, more than AVG, and symptom reduction would be associated with, putatively mediated through, improved VO_2_max.

**Results:**

Depressive symptoms (baseline score 3.97, 95% *CI:* 3.41, 4.52), were −1.03 points more reduced in HIIT than AVG at post-intervention (95% *CI:* −1.71, −0.35, *p* = 0.003), corresponding to a small to moderate effect size (*d* = 0.37) and persisting at follow-up. There was a small reduction in overall symptoms, but no significant between-group differences were observed. Change in VO_2_max correlated negatively with the change in depressive symptoms. Mediation analysis showed a significant effect of change in VO_2_max on change in depressive symptoms within HIIT. The total effect was moderated by group, and depressive symptoms were more reduced in HIIT. Direct effects, not mediated through VO_2_max, were non-significant. Indirect effects, mediated through VO_2_max, were non-significant, but the moderated mediation test indicated a non-significant trend of 0.4 points (95% *CI:* −1.188, 0.087) and a larger reduction in depressive symptoms through VO_2_max in HIIT.

**Conclusion:**

HIIT reduced depressive symptoms more than AVG, which persisted at follow-up. HIIT may serve as a complementing treatment option targeting these symptoms in individuals with schizophrenia, even before they reach clinical depression. Depressive symptoms are important to prevent, stabilize, and treat due to their negative implications for psychological wellbeing and long-term functional outcome. Reduction in depressive symptoms was associated with improved VO_2_max, and non-significant trends in the data supported that improved VO_2_max may be part of the complex mechanisms underlying the anti-depressive effect of HIIT.

**Clinical Trial Registration:**

[www.ClinicalTrials.gov], identifier [NCT02205684].

## Introduction

High-intensity interval training (HIIT), consisting of short intervals of high-intensity work alternating with recovery periods of lower intensity (1), shows beneficial health effects across non-clinical and clinical populations throughout the lifespan, including improved cardiorespiratory fitness (CRF) and mental health (2, 3). However, few studies have investigated the efficiency of HIIT in reducing psychotic and non-psychotic psychiatric symptoms in individuals with schizophrenia (2, 4, 5), and whether an exercise-induced reduction in symptoms is associated with improved CRF.

Schizophrenia involves not only a spectrum of psychiatric symptoms, such as the hallmark positive and negative psychotic symptoms (Diagnostic Manual of Mental Disorders, DSM-IV), but also emotional disturbancies (6). While positive symptoms are responsive to antipsychotic medication, negative symptoms are difficult to treat (7–9). Depressive symptoms are recognized as distinct symptoms in schizophrenia and are frequent both in acute and chronic phases (10). Despite this finding, they remain inadequately recognized or treated, and research on interventions targeting depressive symptoms in schizophrenia is sparse (11). Both negative and depressive symptoms are associated with lower psychological wellbeing (12) and poorer long-term functional outcome (11, 13, 14). Consequently, it is important to find new treatment options targeting these symptoms specifically or overall symptom load more generally.

To our knowledge, three intervention studies have investigated the effect of HIIT on schizophrenia symptoms. A non-controlled pre–post-study including inpatients with chronic schizophrenia by Wu et al. reported a reduction in overall symptoms, and more specifically in general, negative and depressive symptom dimensions but not in positive symptoms, following 8 weeks of supervised HIIT (15). A non-randomized controlled trial by Heggelund et al., comparing 8 weeks of supervised HIIT to Tetris video gaming in inpatients with schizophrenia, described no changes in overall positive, negative, general, or depressive symptoms (16). A non-observer-blinded randomized controlled trial (RCT) by Romain et al. described a significant reduction in negative symptoms, but no significant changes in positive or general symptoms, following 6 months of supervised HIIT in individuals with psychosis and who are overweight compared to waiting list controls. Overall symptoms or depressive symptoms were not reported (17). An anti-depressive effect of HIIT is well-documented in individuals with major depressive disorder (MDD) (5, 18), and is also shown in a meta-analysis encompassing both studies of MDD and psychotic disorders (4). Noteworthy, only two small pre- post-studies on individuals with schizophrenia contributed to the meta-analysis (15, 19).

Physical exercise is described to have positive effects on complex neurobiological processes, such as structural plasticity, upregulation of neurotrophins (i.e., brain-derived neurotrophic factor), modulation of synaptic plasticity, regulation of neurotransmitters, HPA–axis regulation, and immunological mechanisms, potentially targeting pathophysiological aspects of schizophrenia (20). The effect of exercise on mental health is potentially underpinned by these complex neurobiological processes (21–25). It is suggested that improved CRF (as measured by VO_2_max) may induce favorable vascular adaptations and improve cerebrovascular function and cerebrovascular reserve, facilitate the abovementioned neurobiological processes, and thereby be linked to mental health effects of exercise (26). HIIT is a physical exercise mode that is recognized to improve CRF (27) and has been shown to be more efficient than moderate-intensity continuous training (28). Hence, HIIT is suggested to have greater potential than moderate-intensity continuous training for improving psychiatric symptoms (29). HIIT has been shown to improve VO_2_max (30, 31) and reduce symptoms (17) in individuals with schizophrenia, but the relationship between VO_2_max and symptom reduction is not established.

To sum up, the efficiency of HIIT in reducing symptoms in schizophrenia appears promising (15, 17), albeit not sufficiently investigated. It is too early to conclude whether HIIT may reduce overall symptoms or exhibit more specific effects on symptom domains. It is of special interest to investigate if the anti-depressive effect of HIIT, shown in individuals with MDD and in a pre–post-study on inpatients with schizophrenia, may also be found in a methodological more robust study on individuals with schizophrenia. Furthermore, it is of interest to analyze whether symptom reduction following HIIT is associated with, or putatively partly mediated by, increased VO_2_max.

The main aim of the current study was to investigate the efficiency of HIIT in reducing psychotic and non-psychotic symptoms in schizophrenia, and whether a reduction in symptoms was associated with improvement in VO_2_max. To account for treatment effects due to supervision in the HIIT group, the comparison group performed supervised low-intensity (32) active video gaming (AVG) consisting of computerized sports simulation, intended to control for social interaction and time spent. We complement previous HIIT studies in schizophrenia with a larger and observer-blinded RCT, adding follow-up assessment to look for the persistence of effects. We applied a single outcome measure covering both psychotic and non-psychotic symptoms on the same metric to avoid method-specific effects. The factor analytically derived five-factor model of PANSS (33) provides an empirically based differentiation of a positive, negative, excited, disorganized, and primary interest for this study, a depressed symptom factor. We applied all five factors and also the Calgary Depressive Symptoms Scale for schizophrenia (CDSS) for the additional assessment of depressive symptoms (34). Because HIIT was investigated in individuals prescribed antipsychotic medication, we were reluctant to expect reductions in positive symptoms beyond the effect of medication. No previous HIIT studies have shown an effect on positive symptoms. The effect of HIIT on negative symptoms has been shown in one non-observer-blinded RCT (17), but was not found in a comparable non-randomized controlled trial (16). Due to these inconsistent results and because negative symptoms are recognized as difficult to treat (8, 35), we did not expect a reduction in negative symptoms, albeit recognizing the clinical importance of targeting these symptoms. To our knowledge, no previous studies have investigated the effects of HIIT on excited or disorganized symptoms.

First, we hypothesize that HIIT reduces overall symptoms more than AVG, and in particular that HIIT reduces depressive symptoms more than AVG. Second, we hypothesize that reduction in symptoms is associated with improved VO_2_max, and putatively that VO_2_max mediates the effect of HIIT on symptoms.

## Materials and methods

The EPHAPS trial is pre-reported in Clinical Trials (NCT02205684, 31/07/2014) and approved by the Regional Committees for Medical and Health Research Ethics (trial number 2014/372). The method followed and the materials used are more thoroughly described in the study protocol (36). We previously reported a sports physiological study on cardiorespiratory effects in the EPHAPS RCT (37), describing no significant between-group differences in VO_2_max change and no within-HIIT group increase in mean VO_2_max, despite documenting engagement in HIIT by attendance to sessions and intervals, and by heartrate assessments during HIIT sessions (37). However, a 5% or higher increase in VO_2_max was found in 47% of the HIIT group compared to 27% of the AVG group, and the HIIT group showed a significant increase in workload (heart rate, treadmill speed, and inclination). When adding the physical exercise competence of the supervisors to the model, the HIIT group showed a significantly larger increase in mean VO_2_max than the AVG group (post-hoc analyses). This study only included participants with valid VO_2_max defined by respiratory exchange ratio (RER) ≥ 1.0 based on pre- to post-intervention time points (*n* = 47). The current study presents an intention-to-treat (ITT) analysis of all available VO_2_max data from all time points (*n* = 82). We previously reported the primary outcome, neurocognition, according to CONSORT guidelines (38) (showing the Consort diagram of EPHAPS), suggesting neurocognitive improvements following HIIT and AVG (39). The current study reports the secondary outcome of EPHAPS, pre-defined as psychotic and non-psychotic symptoms.

### Study sample, attrition, and protocol violation

The participants eligible for the study were outpatients fulfilling the criteria for schizophrenia according to the Diagnostic and Statistical Manual of Mental Disorders (5th edition), confirmed by the Structured Clinical Interview for DSM-IV axis 1 disorders (SCID-1) (40). Inclusion criteria were age 18–67 years and understanding and speaking a Scandinavian language. Exclusion criteria were comorbid diagnosis of mild intellectual disability, pregnancy, or medical conditions incompatible with participation in physical exercise (36). Patient recruitment and interventions were carried out by the intervention staff. Participants were recruited from two outpatient clinics at Vestfold Hospital Trust. Participation was based on informed and written consent and did not interfere with ongoing pharmacological or non-pharmacological treatment as usual (TAU). The intervention period was extended by one semester to increase the sample size and was conducted from August 2014 through May 2017, with data collection ending in September 2017. Blinded researchers performed the assessments, except for VO_2_max assessments that were performed by a physiotherapist and sport-educated nurse from the intervention staff in collaboration with a sport-physiologist. The psychiatric outcome assessments were performed by assessors blinded for group allocation (two psychiatrists, reliability rated on SCID-I and PANSS). Eighty-two participants were included in the study and randomly allocated to HIIT (*n* = 43) or AVG (*n* = 39) using a computerized random block generator (stratified by VO_2_max), concealed envelopes, and a remote study coordinator.

Participant flow in the HIIT group: Forty-three participants were allocated to the HIIT group and were included in the efficiency analyses run as ITT. Four dropped out before intervention and five discontinued intervention, and thus 34 completed post-intervention testing. Five were lost to follow-up, and 29 completed follow-up assessments. Four of these had protocol violations, and thus 25 were included in the efficacy analyses run as per protocol (PP) (an additional eight had protocol violations but also dropped out before completing the study).

Participant flow in the AVG group: Thirty-nine participants were allocated to the AVG group and were included in the ITT analyses. One dropped out before intervention and one discontinued intervention, and thus 37 completed post-intervention assessment. Nine were lost to follow-up, and 28 completed follow-up assessment. All 28 were included in the PP analyses (two participants in the AVG group had protocol violation but also dropped out before completing the study).

For more information, including reasons for drop out and type of protocol violation, and the consort diagram, we refer to Bang-Kittilsen et al. (39).

### Interventions

The HIIT intervention consisted of treadmill walking/running following a standardized program consisting of 8 min of warm-up, followed by 4 × 4 min high-intensity intervals at 85–95% of the maximal heart rate (HRmax) (peak heart rate from the VO_2_max test was used to prescribe individualized intensity) alternating with 3 min of active recovery phases (walking/running) at ∼70% HRmax, and finally 5 min of cool down (37).

The comparison active video group (AVG) performed computerized interactive sport simulation (Nintendo-Wii, sport). Each session lasted 45 min. Moving the body while holding a remote control initiated a sport simulation on a screen. Each participant could each time choose between a simulation of bowling, golf, or tennis.

Intensity level, or cardiorespiratory demands, was aimed to be the main difference. Both HIIT and AVG had a frequency of two sessions per week and a duration of 12 weeks, were continuously supervised by the same intervention team consisting of sport and/or mental health educated employees trained to supervise both interventions, and given individually or in groups of 2–3 participants remote from were assessments were performed.

Attendance to sessions (*n* = 24) was median 18/mean 15.70 [standard deviation (*SD*): 7.34] in the HIIT group and median 20/mean 18.28 (*SD:* 4.63) in the AVG group. For protocol-compliant study completers, the mean attendance was median 20/mean 19.96 (*SD:* 2.42), range 0–24, in HIIT and 20/19.39 (*SD:* 1.93), range 0–24, in AVG. Within the HIIT group, the mean attendance to the 96 possible high-intensity intervals (four per session) was 70 (*SD:* 19).

### Assessments

Symptoms were blindly assessed at baseline, post-intervention, and 4 months of follow-up using the PANSS, which is known to show good psychometric properties, reliability, validity, and sensitivity (41). We shifted the interval item scale (1–7) to the ratio version scale (0–6) to avoid the bias induced by a minimum value different from zero and to make the interpretation easier (42, 43). Overall symptoms were calculated by summarizing all 30 items (PANSS total), and we investigated our hypothesis of specific effects on symptoms by using a five-factor model (33) with 20 items distinctly distributed to five factors (described in note, Table 1).

**TABLE 1:** Observed demographic and clinical baseline characteristics (*n* = 82).

	High-intensity interval training (*n* = 43)					Active video gaming (*n* = 39)				
Parameter	Median	Mean	(*SD*)	95% *CI*	%	Median	Mean	(*SD*)	95% *CI*	%
Age (years)	31	36.6	(14.3)	32.2, 41.0		33	37.5	(13.8)	33.0, 42.0	
Gender (%men)					61					62
WAIS-IV GAI^†^	87.0	85.8	(14.2)	81.5, 90.2		90.0	89.0	(16.9)	83.5, 94.5	
MCCB^‡^	33.5	32.8	(8.4)	30.2, 35.3		34.7	35.2	(8.4)	32.5, 38.0	
GAF^§^ function	43.0	42.2	(7.4)	40.0, 44.5		45.5	45.7	(7.9)	43.1, 48.3	
GAF^§^ symptoms	41.0	41.6	(7.6)	39.2, 43.9		42.5	44.5	(8.2)	41.8, 47.1	
Duration of illness	13.0	12.9	(10.3)	9.5, 16.4		11.0	14.6	(12.6)	10.3, 18.9	
PANSS^¶^ total (0–180)	38.0	38.5	(16.9)	33.2, 43.7		34.0	34.1	(16.0)	28.8, 39.3	
PANSS positive (0–24)	6.0	6.0	(3.9)	4.7, 7.2		4.0	5.1	(4.2)	3.7, 6.5	
PANSS negative (0–36)	10.0	10.1	(7.2)	7.8, 12.4		8.0	8.5	(6.0)	6.5, 10.4	
PANSS disorganized (0–18)	5.0	5.0	(2.7)	4.1, 5.8		3.0	3.9	(3.3)	2.8, 5.0	
PANSS excited (0–24)	1.0	1.8	(1.9)	1.2, 2.4		1.0	2.1	(2.5)	1.3, 2.9	
PANSS depressed (0–18)	4.0	4.2	(2.6)	3.3, 5.0		4.0	3.7	(3.0)	2.7, 4.7	
CDSS^††^	2.0	3.5	(3.5)	2.4, 4.5		2.0	3.1	(3.6)	2.0, 4.3	
Antipsychotics DDD^‡‡^	1.5	1.9	(1.2)	1.5, 2.2	98	1.5	1.4	(0.7)	1.2, 1.6	95
Antidepressants DDD	0.0	0.3	(0.7)	0.1, 0.5	19	0.0	0.5	(1.1)	0.2, 0.9	28
Mood stabilizers DDD	0.0	0.1	(0.3)	0.0, 0.2	14	0.0	0.1	(0.2)	0.0–0.1	8
VO_2_max ^§§^	28.2	29.9	(11.5)	26.4, 33.5		29.8	29.5	(10.8)	26.0, 33.0	

SD, standard deviation; CI, confidence level; %, proportion.

^†^Wechsler’s Adult Intelligence Scale-Fourth Edition, General Ability Index.

^‡^MATRICS Consensus Cognitive Battery, neurocognitive composite score.

^§^Global Assessment of Functioning, symptoms, and function (missing AVG = 1). Duration of illness (years) (missing HIIT = 7/AVG = 4).

^¶^The Positive And Negative Syndrome Scale, NB! Ratio version (item scale shifted to 0–6). Total = Sum of all 30 items (0–180p) (missing HIIT = 1/AVG = 1). The five-factor model by Wallwork et al. Positive factor: P1 delusion, P3 hallucinations, P5 grandiosity, and G9 unusual thought content (0–24p) (missing HIIT = 3). Negative factor: N1 blunted affect, N2 emotional withdrawal, N3 poor report, N4 passive/apathetic social withdrawal, N6 lack of spontaneity, and G7 motor retardation (0–36p) (missing HIIT = 3). Disorganized factor: P2 conceptual disorganization, N5 Difficulty in abstraction and G11 poor attention (0–18p) (missing HIIT = 3/AVG = 1). Excited factor: P4 excitement, P7 hostility, G8 unusual thought content, and G14 poor impulse control (0–24p) (missing HIIT = 3). Depressed factor: G2 anxiety, G3 guilt feeling, and G6 depression (0–18p) (missing HIIT = 3).

^††^Calgary Depressive Scale for Schizophrenia.

^‡‡^Defined daily doses, calculated in accordance with guidelines from the World Health Organization Collaborating Center for Drug Statistics Methodology (http://www.whocc.no/atcdd).

^§§^Maximum oxygen uptake (missing HIIT = 1).

CDSS was applied for additional assessment of depressive symptoms (34). CRF (VO_2_max) was measured at all three time points assessed in a treadmill-based maximum exercise test using a modified Balke protocol (44), which is more thoroughly described in Andersen et al. (37).

For the current purpose, an assessment of the General Ability Index (GAI) of the Wechsler Adult Intelligence Scale, fourth edition (45) and the MATRICS Consensus Cognitive Battery (46, 47) was performed to assess baseline cognitive functioning. The Global Assessment of Functioning Scale (GAF) was used to describe baseline symptoms and function (48). Information about the duration of illness and pharmacological treatment was gathered from interviews and medical records.

The psychotropic drugs prescribed for the participants were presented as a defined daily dose (DDD) based on approved dose recommendations. DDD provides a rough estimate of participants’ drug consumption utilizing the assumed average maintenance dose per day for each specific drug used independently of the dosage form for its main indication in adults (i.e., schizophrenia for antipsychotics). For example, the DDDs for chlorpromazine and risperidone are 300 and 5 mg, respectively. DDD was calculated in accordance with the guidelines provided by the World Health Organization Collaborating Center for Drug Statistics Methodology.^1^

### Statistical analyses

Following the Consort guidelines and due to randomization, significance testing at baseline was not performed, but average values and variance for the two groups are reported in Table 1 (49).

#### Primary analysis of symptoms and VO_2_max: Mixed-effects models, intention to treat

Independent and similar analyses were conducted for the total PANSS score (overall symptoms) and for each of the five factors. Mixed-effects models were used to assess the effects of the variables of interest (time, time and group interaction at post-intervention, and time and group interaction at follow-up) while accounting for correlation due to multiple observations within participants. Analyses were run with and without age and sex in the model to confirm the consistency of the results. Mixed-effects models utilize all available data. The analyses were conducted following the ITT principle. Missing values reduce the precision of the estimates and may potentially also induce bias. The type of missing was analyzed using Little’s missing completely at random test (50). To confirm the ITT results and to analyze the efficacy of the interventions, PP analyses of protocol-compliant study completers were employed.

The analysis proceeded by fitting a set of models that reflected the clinical questions of interest. In particular, the interest focused on two issues: the course of symptoms (PANSS scores) over the span of the observation period in the two groups and the potential differences in levels and trends between the two groups. All models were constrained to equal response at baseline for the two groups, in accordance with initial testing. Hence, baseline values for the two groups were represented by the intercept of the model. By design and with only three time points, the data were not amenable to fitting models with continuous time effects. Consequently, time was treated as a factor variable (post-intervention and follow-up) with baseline as a reference. The main effect of the group was not included in the model, but different courses over time for the two groups were accommodated by interaction terms between groups and time points. The base model allowed for free variation over both time and group and served as an offset for sub-models that tested no change from baseline to post-intervention or follow-up or both, independently for each group, as well as sub-models that tested no difference between the two groups at each and all time points. These constituted a succession of nested sub-models, all rooted in the base model. The likelihood ratio test was used to remove insignificant effects and thereby reach our best fit final models. Specific models were built to test hypotheses on persistent effects, i.e., a potential change from baseline to post-intervention but no change from post-intervention to follow-up. This could be at the same level or at different levels for the two groups. These models were not sub-models of the base model and therefore not reached by reductions from that. Instead, we used Akaike’s information criterion (AIC) for model selection.

Standardized residuals were inspected and confirmed for normality by quantile normal plots. Heterogeneity of variance was inspected using scatterplots of standardized residuals and fitted values.

Effect sizes are not given by mixed-effect models, but recommended to report (38). The estimated between-group differences in change (Table 2) were divided by baseline standard deviation in the sample (Table 1), interpreted according to guidelines for Cohen’s d *(d)* (51), and used to characterize the magnitude of the between-group effects. For overall symptoms (total PANSS), the relative change in total PANSS was calculated.

**TABLE 2:** Efficiency analyses, mixed-effects model, and intention-to-treat protocol, best fit models.

	Parameter	Description of change	Effects	Estimates (*SE*)	95% *CI*	*P-value*
Overall symptoms	PANSS total^†^	Reduction in both groups, but no group difference	Baseline	36.32 (1.82)	(32.76, 39.89)	
			Post-intervention	−3.29 (1.39)	(−6.01, −0.58)	0.018
			Follow up	−4.16 (1.52)	(−7.14, −1.18)	0.006

Psychotic and non-psychotic symptoms	PANSS positive^‡^	Reduction in both groups, but no group difference	Baseline	5.64 (0.45)	(4.76, 6.52)	
			Post-intervention	−0.51 (0.35)	(−1.20, 0.19)	0.153
			Follow up	−1.09 (0.39)	(−1.85, −0.34)	0.005
	
	PANSS negative ^‡^	Stability in both groups	Baseline, post-intervention and follow-up	9.05 (0.70)	(7.68, 10.43)	
	
	PANSS Disorganized^‡^	Stability in both groups	Baseline, post-intervention and follow-up	4.51 (0.30)	(3.93, 5.09)	
	
	PANSS Excited^‡^	Stability in both groups	Baseline, post-intervention and follow-up	1.87 (0.20)	(1.47, 2.26)	
	
	PANSS Depressed^‡^	No change in AVG, significant reduction in HIIT compared to AVG at post-intervention, relative to baseline, persisting to follow-up	Baseline	3.97 (0.28)	(3.41, 4.52)	
			Group × time^§^	−1.03 (0.35)	(−1.71, −0.35)	0.003

Depressive symptoms	CDSS^¶^	No change in AVG, significant reduction in HIIT compared to AVG at follow-up, relative to baseline	Baseline	1.12 (0.8)	(0.95, 1.28)	
			Group × time at post-intervention	−0.13 (0.13)	(−0.39, 0.13)	0.320
			Group × time at follow-up	−0.31 (0.14)	− 0.59, −0.03)	0.032

Cardio- respiratory fitness	VO_2_max^††^	No change in HIIT or AVG at post-intervention. Reduction in HIIT compared to AVG at follow-up relative to baseline	Baseline and post-intervention	29.91 (1.16)	(27.64, 32.19)	
			Group × time at follow-up	−2.37 (0.74)	(−3.82, −0.92)	0.001

SE, Standard error; CI, confidence level. Group 0 = AVG, 1 = HIIT. Post-intervention and follow-up are both compared to baseline.

^†^The Positive And Negative Syndrome Scale, ratio version (item scale shifted to 0–6). Total = Sum of all 30 items (0–180p).

^‡^PANSS symptom factors from the five-factor model by Wallwork et al. (33), for more details see Table 1.

^§^Post-intervention and follow-up were merged into a single time point (time after baseline) as no changes were detected in either group between these two time points.

^¶^Calgary Depression Scale for Schizophrenia, transformed by adding 1 to facilitate logarithmic transformation, and log-transformed (ln).

^††^Maximum oxygen uptake (missing HIIT = 1).

Non-significant effects are taken out of the models. Supplementary Table 1 shows the more saturated models, including both time and interaction effects of time and group.

#### Secondary analysis of the relationship between VO_2_max and symptoms: Moderated mediation analysis

We first explored the relationship between change in VO_2_max and change in symptoms by correlation analysis (Pearson).

To investigate VO_2_max as a putative underlying mechanism more thoroughly and whether this was moderated by group, we performed a moderated mediation analysis [MEMORE (52), model 4, using SPSS version 26]. Post- and pre-intervention levels of depressive symptoms were included in the model as outcome, post- and pre-intervention levels of VO_2_max as a mediator, and group as a moderator. The number of bootstrapping iterations was set to 5,000 (default). Participants with missing data were excluded from this analysis, as the MEMORE macro does not allow for missing data. The macro allows for only two time points.

## Results

### Baseline descriptives

The demographic and clinical characteristics are presented in Table 1. For the total sample (*n* = 82), the mean empirical overall symptom score on PANSS reflects mild to moderate symptom severity (53). The mean scores on PANSS depressed factor and CDSS reflect mild depressive symptoms, albeit 21/82 participants had a score ≥ 6, indicating clinical depression.

Participation in the study did not interfere with the clinical treatment. Randomization aims to control for confounders, such as medication. Larger sample size would allow for including medication in the models. An inspection of the data did not reveal large differences between the two groups. See Table 1 for baseline mean defined daily doses (DDDs) of the three main groups of medication (antipsychotics, antidepressants, and mood stabilizers) in HIIT and AVG. Ninety-six percent were prescribed antipsychotic medication (*n* = 37/39 in AVG; *n* = 42/43 in HIIT). Observed values of DDD antipsychotic medication in the HIIT group and in the AVG group were stable throughout the three study time points (baseline, post-intervention, and follow-up), respectively. From baseline to post-intervention, seven showed reduced DDD and five increased DDD in the AVG group. In the HIIT group, six showed reduced and five increased DDD. Twenty-three percent were prescribed antidepressants (AVG *n* = 11/39, HIIT *n* = 8/43). From baseline to post-intervention, four showed increased DDD in AVG and one increased DDD in HIIT. Eleven percent were prescribed mood stabilizers (AVG *n* = 3/39, HIIT *n* = 6/43), and one showed reduced and one increased dose in AVG, while two showed increased dose in HIIT.

### Missing data

The outcome data (PANSS) showed 16–18% missing, thereof some intermittent (Table 1) but mainly missing due to attrition. Little’s MCAR test for the hypothesis that data are missing completely at random could not be rejected (including the PANSS variables, group, age, and gender) (*p* = 0.26). Mixed-effects models utilize all available observations, and we chose not to impute the missing values. PP analyses were applied to confirm the robustness of the ITT results.

### Efficiency analyses

**FIGURE 1:**
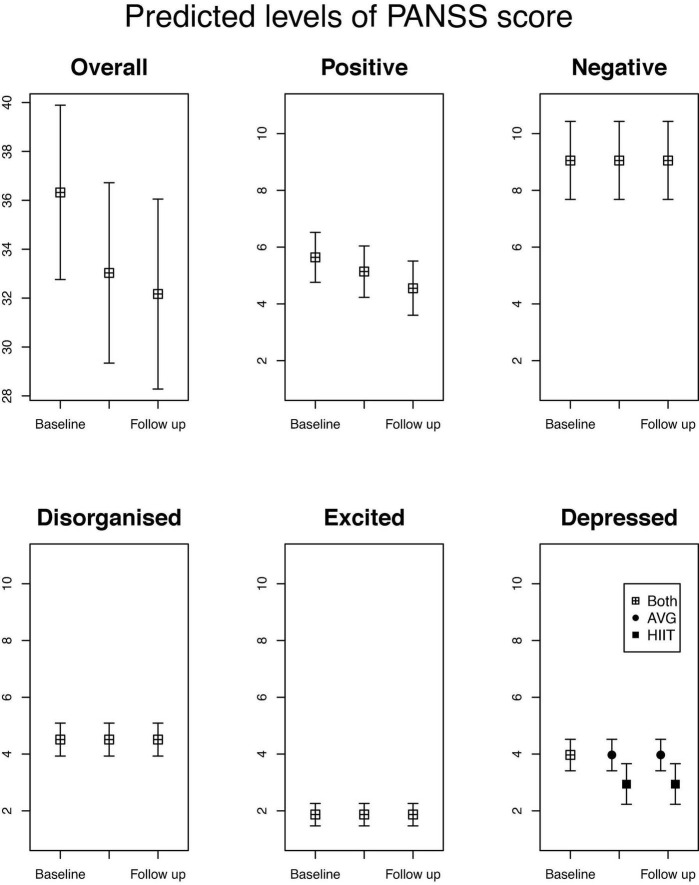
Predicted levels of PANSS score, total and sub-dimensions with 95% confidence levels. For each of comparisons the 5 sub-simensions have been plotted on the same scale.

For the mixed-effects model analysis, the reference group is AVG (AVG = 0/HIIT = 1). The ITT analysis of the best fit models from the mixed-effect models is presented in Table 2 and described below. For the transparency of the analyses, results from the most saturated model (time, time × group at post-intervention, and time × group at follow-up) and the main tested models when taking non-significant effects out of the models until the best fit model is reached are presented in Supplementary Table 1. For visual graphs showing predicted treatment effects (level of PANSS score with 95% CI) and changes in PANSS scores obtained from the estimated best fit models see Figure 1.

The analysis of depressive and overall symptoms was hypothesis-driven, but the remaining symptom factors and VO_2_max were data-driven searching for the best fit model. The results indicated three different pathways of change for the symptoms:

(1) In line with our hypothesis, there was significant between-group differences in depressive symptom reduction over time, with a significantly larger reduction in depressive symptoms at post-intervention in the HIIT group, persisting at follow-up, but no change in the AVG group. The depressed factor was estimated to have 3.97 points (95% *CI:* 3.41, 4.52) at baseline in the total sample. In the HIIT group, depressive symptoms decreased by −1.03 points (95% CI: −1.71, −0.35) more than in the AVG group. The between-group difference in reduction was significant and persisted at follow-up, giving the same estimate at follow-up for the HIIT group. In the AVG group, there was no significant change, and the mean score was estimated to remain stable at 3.97 points at baseline, post-intervention, and follow-up. The between-group difference in the reduction of depressive symptoms corresponded to a small to moderate effect size of *d* = −0.37 (95% *CI:* −0.62, −0.13), *p* = 0.003.

To confirm consistency in results and make the results more robust, parallel analyses were performed using CDSS. CDSS was rescaled by adding 1 and log-transformed [CDSS (ln)]. CDSS (ln) correlated positively with PANSS depressed factor at all-time points, with baseline ρ_*All*_ 0.7, *p* = < 0.001 (*n* = 79), post-intervention ρ_*All*_ 0.8, *p* < 0.001 (*n* = 70), and follow-up ρ_*All*_ 0.7, *p* < 0.001 (*n* = 55), respectively. Change in PANSS depressed factor and change in CDSS (post- to pre-intervention) correlated positively, ρ_*all*_ 0.6, *p* = 0.001. To enable direct comparison with PANSS depressed factor, we also tested the best fit model for PANSS depressed score on CDSS (ln). This model estimated a baseline of 1.12 points (*SE* 0.08) (95% *CI:* 0.95, 1.28), with a non-significant reduction in HIIT compared to AVG at post-intervention that persisted at follow-up [−0.21 (*SE* 0.12) (95% *CI:*−0.43, 0.02), *p* = 0.067]. In the best fit model for CDSS (ln) baseline was estimated to be 1.12 (95% CI: 0.95, 1.28) for both groups. The AVG group was estimated to remain stable at 1.12 points at baseline, post-intervention, and follow-up. CDSS (ln) score in the HIIT group was reduced by −0.13 points at post-intervention compared to AVG, not reaching significance, but was significantly more reduced in the HIIT group compared to the AVG group at follow-up [−0.31 points (95% *CI:* −0.59, −0.03)] relative to baseline. See Supplementary Table 1 for the main tested models.

(2) For overall and positive symptoms, there was a significant symptom reduction over time, but no significant between-group differences. Contrary to our hypothesis, the interaction effect of group and time was not significant for overall symptoms, but a significant effect of time was noticed in this study. Overall symptom load at baseline was estimated to be 36.32 (95% *CI:* 32.76, 39.89) and decreased by −3.29 points (95% *CI:* −6.01, −0.58), at post-intervention and by −4.16 points (95% *CI:* −7.14, −1.18) at follow-up in both groups. This corresponds to a 9 and 12% reduction from baseline. The positive factor [estimated baseline score 5.64 (95% *CI:* 4.76, 6.52)] was estimated to remain stable post-intervention, but to decrease by −1.09 points (95% *CI:* −1.85, −0.34) at follow-up in both groups, preceded by a non-significant decreasing trend at post-intervention.

(3) Estimations for negative, excited, and disorganized symptoms indicated symptom stability over time and no between-group differences. The disorganized factor was estimated to remain stable at 4.51 points (95% *CI:* 3.93, 5.09) in both groups at all three time points. The excited factor was estimated to remain stable at 1.87 points (95% *CI:* 1.47, 2.26) in both groups at all-time points. The negative factor was estimated to remain stable at 9.05 (95% *CI:* 7.68, 10.43) for both groups at all-time points.

VO_2_max was estimated to 29.91 (95% CI: 27.64, 32.19) at baseline for both groups, and was estimated to remain stable at this value at post-intervention for both groups. At follow-up, there was a significant reduction in VO_2_max in HIIT compared to AVG, estimated to be −2.37 (95% CI: −0.82, −0.92) relative to baseline.

To check for the possible confounding effects of age and sex, all analyses were run with and without the main effects of age and sex. The main effect of age was significant for VO_2_max only. Age is expected to affect baseline value, but not change in VO_2_max (54). The main effect of sex was significant for negative symptoms only. The inclusion of age in VO_2_max analysis and sex in the analysis of negative symptoms affected the baseline value, but the estimates for time and time × group interaction were consistent. Analyzing sex differences was beyond the scope of this study.

The hypothesis of equal baseline values was accepted for all parameters. The distributions of the standardized residuals caused no concern for normality or homogeneity of variance.

### Efficacy analyses

The results from the ITT analyses remained robust following PP analyses of compliant completers (*n* = 53). An exception was excited symptoms [baseline 2.2 points (95% *CI:* 1.67, 2.69)], estimated to decrease by −0.7 points (95% *CI:* −1.12, −0.20), *p* = 0.005, at follow-up, preceded by a non-significant decreasing trend of −0.4 points (95% *CI:* −0.90, 0.01), *p* = 0.056, at post-intervention in compliant completers.

### Secondary analysis of the relationship between change in VO_2_max and depressive symptoms

#### Correlations between change in VO_2_max and change in depressive symptoms

The differences in the scores (post-intervention and baseline) were calculated. There was a negative Pearson correlation between change in VO_2_max and change in overall symptoms (ρ_*All*_ −0.32, *p* = 0.010; ρ_AVG_ −0.32, *p* = 0.060; ρ_HIIT_ –0.30, *p* = 0.113). There was a negative correlation between change in VO_2_max and change in depressive symptoms (ρ_*All*_ −0.36, *p* = 0.003; ρ_AVG_ −0.20, *p* = 0.244; ρ_HIIT_ −0.45, *p* = 0.013). Change in VO_2_max also correlated negatively with change in CDSS score [ρ_*All*_ −0.37, *p* = 0.002; ρ_*AVG*_ −0.23 (*p* = 0.17); ρ_*HIIT*_ −0.48 (*p* = 0.006)]. There was a negative correlation between change in VO_2_max and change in excited symptoms (ρ_All_ −0.31, *p* = 0.01; ρ_AVG_ −0.38, *p* = 0.02; ρ_HIIT_ –0.30, *p* = 0.109). This indicated an association between increase in VO_2_max and reduction in these symptoms. Significant correlations were not found for change in VO_2_max and change in positive (ρ_*All*_ −0.14, *p* = 0.264), negative (ρ_*All*_ −0.20, *p* = 0.105), or disorganized symptoms (ρ_*All*_ −0.05, *p* = 0.699).

#### Moderated mediation analysis for repeated measure designs

Moderated mediation analyses were only performed on PANSS depressed factor data, as it was deemed normally distributed and highly correlated with CDSS. The total effect on VO_2_max was not significant [−0.30 (95% *CI:* −1.62, 1.03), *p* = 0.659] and group did not significantly moderate the effect [1.57 (95% *CI:* −0.43, 3.58), *p* = 0.121]. Thus, there was neither a significant within-group change in VO_2_max in AVG [−0.30 (95% *CI:* −1.62, 1.03), *p* = 0.659] nor HIIT [1.28 (95% *CI:* −0.22, 2.78), *p* = 0.093]. These results (*n* = 66) are in line with both the current mixed-effects model ITT analysis (*n* = 82) and with the previously published subset (*n* = 47) analysis by Andersen et al. that excluded all VO_2_max assessments with RER < 1.0 to ensure valid assessment (37). The main results are presented in Table 3.

**TABLE 3:** Moderated mediation regression analysis for repeated measure designs (*n* = 66).

PANSS depressed factor	*Conditional total, direct and indirect effects*							*Indices of moderation by group*				
		
Effects	Moderator (Group)	*Mediator Average (VO_2_max)*	Effect	SE	LLCI	ULCI	p	Effect	SE	LLCI	ULCI	*P*
Total effects	AVG		0.16	0.39	−0.62	0.95	0.682	−1.23	0.59	−2.42	0.05	0.042
	HIIT		−1.07	0.45	−1.96	−0.18	0.019					
Direct effects	AVG	−0.26	0.13	0.36	−0.59	0.85	0.721	−0.84	0.56	−1.95	0.27	0.138
	HIIT	0.32	−0.70	0.42	−1.55	0.15	0.103					
Indirect effects^†^	AVG		0.03	0.09	−0.16	0.22	−	−0.40	0.32	−1.19	0.09	−
	HIIT		−0.37	0.31	−1.15	0.07						

Moderated mediation analysis: Outcome (Y) is the difference in depressive symptoms (post-pre-intervention PANSS depressed factor score), moderator (W) is the group (AVG = 0/HIIT = 1), and mediator (M) is the difference in VO_2_max (post-pre-intervention). Effect, Regression coefficient; SE, standard error; LLCI-ULCI, 95% Confidence interval; Total effect, The total conditional effect; Direct effect, The conditional effect not mediated by VO_2_max; Indirect effect, The conditional effect through VO_2_max.

^†^For indirect effects, the standard errors and confidence intervals are bootstrapped.

There was a significant effect of change in VO_2_max on change in depressive symptoms in the HIIT group [−0.29, (95% *CI:* −0.48, −0.10), *p* = 0.004], but not in the AVG group [−0.11 (95% *CI:* −0.30, 0.08), *p* = 0.249]. This group difference was, however, not significant [−0.18 (95% *CI:* 0.45, 0.10), *p* = 0.20].

The *total effect* on depressive symptoms was significantly moderated by group: There was no significant effect in the AVG group, while there was a significant reduction of depressive symptoms in the HIIT group (see Table 2). The *direct effect* on depressive symptoms (the effect that is not explained by VO_2_max) was significant neither for HIIT nor AVG. The *indirect effect* on depressive symptoms through VO_2_max (mediation) was significant neither for AVG nor HIIT. The moderated mediation test, comparing the indirect effects through VO_2_max in the two groups, was not significant, but the bootstrap confidence interval and trends in the data indicated a tendency toward a larger reduction in depressive symptoms through VO_2_max in the HIIT group.

Despite not reaching statistical significance, the moderated mediation test, together with trends in the data, was interpreted to support that improved VO_2_max may be part of the complex mechanisms underlying the anti-depressive effect of HIIT.

## Discussion

This is, to the best of our knowledge, the first observer-blinded RCT on individuals diagnosed with schizophrenia to report that HIIT may reduce depressive symptoms significantly more than AVG, to show that this effect persists for 4 months after the intervention and that reduction in depressive symptoms is associated with improved VO_2_max.

The current results are in line with the anti-depressive effect of HIIT shown in studies of individuals with MDD (5), and suggest that HIIT may also reduce depressive symptoms in individuals with schizophrenia. The magnitude of change was small to moderate, and comparable to the effects of antidepressants (55). The results are incongruent with Heggelund et al. (16), also using CDSS as an outcome measure for depressive symptoms, but not directly comparable due to sample differences and statistical analyses. Our results are in line with Wu et al. (15), and are now shown in a methodological more robust design. They are also in line with the meta-analysis on anti-depressive effects of HIIT across mental illnesses by Korman et al. (4).

Finding efficient treatment options with persisting beneficial effects on depressive symptoms in schizophrenia has important clinical implications. The prevalence of depressive symptoms in individuals with schizophrenia range from 25 to 81%, and the reduction of depressive symptoms is pivotal to psychological wellbeing and has favorable implications for a long-term functional outcome (11, 14). Conley et al. found that maintenance of non-depressed status was associated with a reduced need for inpatient and emergency mental health care services, an increase in working days and participation in common activities, and improved general life satisfaction (14). Changing from non-depressed to depressed or vice versa was associated with an improvement or worsening of the functional outcome, respectively. Pharmacological or psychological therapy are important treatment options. In the current sample, the mean level of depressive symptoms was mild and indicates that HIIT may complement existing treatments by targeting mild depressive symptoms before reaching clinical depression. Putatively, HIIT may also target physical comorbidities associated with depression in schizophrenia (21, 56), amongst others by improving risk factors, such as sedentary time and low CRF (57, 58). HIIT is feasible in individuals with schizophrenia as long as supervision is provided (31, 59), also as part of long-term clinical treatment (31). Physical exercise is described to show beneficial effects on symptoms in both schizophrenia and affective disorders and is recommended as part of multimodal intervention programs (60).

We also found some reduction in overall symptoms in the total sample, but contrary to our hypothesis, there was no significant difference between HIIT and AVG. This finding was also inconsistent with Heggelund et al., who reported no significant between- or within-group changes in overall symptom load following HIIT or Tetris video gaming (16). However, Wu et al. described a pre–post-reduction in overall symptoms following HIIT (15). In terms of AVG, there is an emerging interest in its effect on symptoms in schizophrenia (61, 62), hitherto focusing on neurocognitive symptoms. In a previous study, we reported neurocognitive improvements following both HIIT and AVG (39). The current results indicate that both HIIT and AVG may have the potential to reduce overall symptoms, which is incongruent with a previous feasibility study and quasi-experimental trial reporting no significant change in psychotic symptoms when comparing AVG to treatment as usual (63). AVG may encompass different modes of exercise, ranging from HIIT to motor coordination training, and further investigation is needed. In terms of the magnitude of change, the current mean reduction in overall PANSS score was 3 points at post-intervention and 4 points at follow-up, which appear to be comparable to ITT results in a study on HIIT and in a study on a combined form of physical exercise (15, 64). This corresponded to a 9–12% change in the current study. The level of change needed to be recognized as a clinically significant change is described as 25–50% in pharmacological research (53). Hitherto, the effect of exercise on symptoms in schizophrenia has been investigated in outpatient and inpatient samples with ongoing TAU, consisting of psychopharmacological (mostly antipsychotics) and non-pharmacological treatments. Hence, the add-on effect of the exercise could be defined as the complementary effect beyond the effect of TAU. We did not find comparable studies on the efficiency of HIIT in reducing symptoms in drug-naïve individuals with schizophrenia.

Unfortunately, our data did not indicate a reduction in negative symptoms following HIIT as reported by Romain et al. (17). Noteworthy, their intervention had a duration of 6 months, and they described a significant within-HIIT group increase in VO_2_max (not measured in the control group). Moreover, their sample included a broader spectrum of disorders (schizophrenia, schizoaffective disorder, bipolar disorder, psychosis not otherwise specified, early psychosis, and MDD), and they compared HIIT to waiting list controls. They described potential biases, such as non-blinded outcome assessment, high attrition rate (59%), and lower global and social functioning in participants who withdrew from the study when compared to the completers. Altogether, these differences and also potential biases may have contributed to the reduced level of negative symptoms. Due to inconsistent findings, the effect of HIIT or other exercise modes on negative symptoms merits further investigation, as it is of clinical importance to find effective treatment options targeting these core symptoms of schizophrenia.

To date, no HIIT study has found beneficial effects on positive symptoms. In the current study, antipsychotic pharmacological treatment was prescribed for 96% of the participants. Antipsychotics are primarily expected to have an effect on positive symptoms, and reduction in positive symptoms beyond the effect of antipsychotics could be difficult to elicit. Our results indicated a small reduction in positive symptoms at follow-up, but no between-group differences. Our data did not indicate a change in disorganized or excited symptoms in the ITT analyses. PP analyses showed a reduction in excited symptoms in compliant completers at follow-up, but no between-group differences. We found no comparable HIIT studies reporting on these symptoms.

In a meta-analysis encompassing physical exercise modes more broadly, improvements in overall positive and negative symptoms were found, but only when extracting four studies that applied aerobic moderate-intensity continuous exercise with session duration over 30 min. It was noted that three HIIT studies appeared not to elicit these effects (65). Romain et al. (17), and the current study, suggest otherwise.

In terms of VO_2_max, our data indicated no between-group differences and no increase in mean VO_2_max within the HIIT group (37). This does not exclude VO_2_max from having a mediating role in the effect of HIIT on depressive symptoms. Interestingly, we found an inverse relationship between change in VO_2_max and change in depressive symptoms from pre- to post-intervention. Mediation analysis is encouraged in RCT studies (66, 67), but demands high statistical power. For smaller samples, bootstrapping methods are recommended (68, 69). Despite not finding a significant mediation through VO_2_max that was moderated by the group, trends in the data were interpreted to support that improved VO_2_max may be part of the complex mechanisms underlying the anti-depressive effect of HIIT.

Studies on the effect of HIIT on depressive symptoms in schizophrenia are sparse, but the current results are encouraging and merit further investigation in a larger sample. The mechanisms underpinning the mental health effect of exercise are not established, but our results indicate that improved VO_2_max may be of importance, presumably as part of a more complex cascade of neurobiological processes (20). Whether HIIT has neurobiological effects that are different from other exercise modes is not sufficiently investigated (29, 70).

The strengths of the current study were well-defined HIIT intervention and a comparison group controlling for time spent and social interaction. The randomization process was undertaken at a site remote from the interventions. The outcome was blindly assessed by trained and reliability-rated psychiatrists, and for the current purpose by applying one outcome assessment method covering both psychotic and non-psychotic symptoms to reduce method-specific effects. We added a 4-month follow-up assessment to investigate the persistence of effects. Attrition was lower than in a comparable RCT (17), but of note, the duration of their intervention was twice as long. The current sample appeared to represent the outpatients diagnosed with schizophrenia both in demographic and clinical respects (Table 1), which facilitates the generalization of the findings beyond the sample. No harm was reported.

The study has limitations. By applying an active comparison intervention, we analyzed the relative effect of HIIT as compared to AVG, and not the absolute effect as compared to a traditional control group. This challenged the interpretation of the lack of between-group differences in overall symptom reduction. The sample size was lower than calculated in the pre-study power analysis, *n* = 126 (36); nonetheless, the sample size was larger than in comparable studies. The sample size could be considered small for mediation analysis, but the bootstrapping method was applied (68, 69). Analyses of overall and depressive symptoms were hypothesis-driven, and Bonferroni corrections for multiple testing were not applied, as they are not recommended when assessing evidence about specific hypotheses (71). We described all tests of significance that were performed, and exact *p*-values were offered. While suggesting an anti-depressive effect of HIIT, we are careful not to conclude about non-significant findings in the mixed-effects model analyses and the moderated mediation analysis due to the sample size. In addition, HIIT was investigated as an add-on intervention to antipsychotic medication, which may affect the potential for symptom change, especially for positive symptoms. Whether depressive symptoms are in fact the only symptom domain responsive to HIIT can therefore not be inferred from the current data.

Future research should investigate whether factors, such as longer duration, higher frequency, exercise mode, or factors associated with individuals, schizophrenia, or lifestyle, moderate the efficacy of HIIT in improving CRF and symptoms (72). Furthermore, an investigation of mechanisms mediating the effect of exercise on symptoms in schizophrenia, such as VO_2_max, neurotrophic factors, and inflammation markers, is warranted. More knowledge about what approach works for whom and why when applying physical exercise as treatment may enable more efficient and personalized treatment.

## Conclusion

The results indicate that HIIT may reduce depressive symptoms, and can serve as a treatment option targeting these symptoms in individuals with schizophrenia. HIIT may complement existing treatment, such as antidepressants, by reducing symptoms even before they reach clinical depression. Reduction in depressive symptoms was significantly associated with improved VO_2_max, and non-significant trends in the data supported that improved VO_2_max could putatively be mediating part of the anti-depressive effect of HIIT. The mechanisms underlying the exercise effects on mental health merit further investigation in a larger sample.

## Data availability statement

The datasets presented in this article are not readily available because data are sensitive in nature and as such the availability is restricted and regulated by Norwegian Laws and EC laws (GDPR). Requests to access the datasets should be directed to JAE (PI), uxjogh@siv.no.

## Ethics statement

The studies involving human participants were reviewed and approved by the Regional Committees for Medical and Health Research Ethics (trial number 2014/372). The patients/participants provided their written informed consent to participate in this study.

## Author contributions

JAE (PI) and JE (Co-PI) initiated the study conception and design. JAE, JE, TH, TB, EA, JM, and GB-K contributed to the data collection and preparation of the database. GB-K, RH, and JE contributed to the statistical analyses in this manuscript. GB-K wrote the manuscript as the first author and prepared the tables. RH prepared Supplementary Figure 1. All authors contributed to and critically reviewed previous versions of the manuscript and approved the final version and contributed to the interpretation of the results.

## Footnotes

1
http://www.whocc.no/atcdd
