# Supplementary material for: High-intensity interval training may reduce depressive symptoms in individuals with schizophrenia, putatively through improved VO2max: A randomized controlled trial
Source: Front Psychiatry. 2022 Aug 4;13:921689. doi: 10.3389/fpsyt.2022.921689 (PMC9394183; doi:10.3389/fpsyt.2022.921689)
Supplement: Supplementary file 1 [file Table_1.pdf]

Table S1 Efficiency analyses, mixed effects models, intention-to-treat: The main models that were tested for each factor, taking non-significant factors out of the model until the best fit models in bold.

Symptoms	Model 1				Model 2				Model 3			
	Effects	*Estimate (SE)*	*95%CI*	*p*	*Effects*	*Estimate (SE)*	*95%CI*	*p*	*Effects*	*Estimate (SE)*	*95%CI*	*p*
Overall	Baseline	36.32 (1.82)	32.75,39.89		**Baseline**	**36.32 (1.82)**	**32.76, 39.89**					
	Time post-intervention	-3.15 (1.87)	-6.82, 0.52	0.093	**Time post-intervention (both groups)**	**-3.29 (1.39)**	**-6.01, -0.58**	**0.018**				
	Time follow-up	-3.86 (2.07)	-7.91, 0.20	0.062	**Time follow-up**	**-4.16 (1.52)**	**-7.14, -1.18**	**0.006**				
	Group×time post-intervention	-0.30 (2.61)	-5.42, 4.82	0.909	**-**							
	Group×time follow-up	-0.62 (2.90)	-6.29, 5.05	0.831	-							
Positive factor[†]	Baseline	5.64 (0.45)	4.76, 6.52		**Baseline**	**5.64 (0.45)**	**4.76, 6.52**					
	Time post-intervention	-0.80 (0.47)	-1.72, 0.12	0.088	**Time post-intervention (both groups)**	**-0.51 (0.35)**	**-1.20, 0.19**	**0.153**				
	Time follow-up	-1.05 (0.53)	-2.08, -0.02	0.046	**Time follow-up (both groups) (both groups)**	**-1.09 (0.39)**	**-1.85, -0.34**	**0.005**				
	Group×time post-intervention	0.63 (0.66)	-0.67, 1.93	0.340	**-**							
	Group×time follow-up	-0.07 (0.73)	-1.50, 1.36	0.925	-							
Negative factor	Baseline	9.31 (0.75)	7.84, 10.78		Baseline	9.30 (0.75)	7.84- 10.77					
	Time post-intervention	0.11 (0.72)	-1.53, 1.31	0.877	Time post-intervention (both groups)	-0.49 (0.54)	-1.55- 0.57	0.365				
	Time follow-up	-0.07 (0.80)	-1.63, 1.50	0.931	Time follow-up (both groups)	-0.37 (0.59)	-1.53- 0.79	0.532	**Baseline, post-intervention & follow-up (stable scores in both groups)**	**9.05 (0.70)**	**7.68,10.43**	
	Group×time post-intervention	-0.80 (1.02)	-2.79, 1.21	0.437	-							
	Group×time follow-up	-0.63 (1.13)	-2.85, 1.58	0.576	-							

Disorganized factor	Baseline	4.42 (0.35)	3.74, 5.10		Baseline	4.42 (0.35)	3.74, 5.09					
	Time post-intervention	0.33 (0.45)	-0.55, 1.20	0.464	Time post-intervention (both groups)	0.13 (0.34)	-0.54, 0.81	0.700				
	Time follow-up	0.27 (0.50)	-0.71, 1.24	0.596	Time follow-up (both groups)	0.21 (0.37)	-0.53, 0.94	0.582	**Baseline, post-intervention & follow-up (stable scores in both groups)**	**4.51 (0.30)**	**3.93, 5.09**	
	Group×time post-intervention	-0.41 (0.61)	-1.60, 0.78	0.502	-							
	Group×time follow-up	-0.13 (0.67)	-1.45, 1.19	0.851	-							
Excited factor	Baseline	1.97 (0.23)	1.52, 2.41		Baseline	1.97 (0.23)	1.52, 2.42					
	Time post-intervention	-1.15 (0.27)	-0.68, 0.39	0.591	Time post-intervention	-0.01 (0.21)	-0.42, 0.40	0.952				
	Time follow-up	-0.68 (0.31)	-1.28,-0.09	0.025	Time follow-up	-0.41 (0.23)	-0.85, 0.04	0.074	**Baseline, post-intervention & follow-up (stable scores in both groups)**	**1.87 (0.20)**	**1.47, 2.26**	
	Group×time post-intervention	0.29 (0.38)	-0.45, 1.03	0.446								
	Group×time follow-up	0.57 (0.42)	-0.25, 1.39	0.173								
Depressed factor	Baseline	3.92 (0.31)	3.32, 4.52		Baseline	3.97 (0.28)	3.41, 4.52		**Baseline**	**3.97 (0.28)**	**3.41, 4.52**	
	Time post-intervention	0.08 (0.38)	-0.67, 0.82	0.844								
	Time follow-up	0.23 (0.42)	-0.60, 1.06	0.585								
	Group×time post-intervention	-1.23 (0.53)	-2.26, -0.20	0.019	Group×time post-intervention	-1.18 (0.40)	-1.97, -0.39	0.003	**Group×time post intervention & follow-up[‡] Significantly larger reduction in HIIT compared to AVG).**	**-1.03 (0.35)**	**-1.71, -0.35**	**0.003**
	Group×time follow-up	-1.05 (0.58)	-2.18, 0.08	0.069	Group×time follow-up	-0.84 (0.43)	-1.68, -0.00	0.050				
CDSS[§] (ln)	Baseline	1.12 (0.09)	0.94, 1.30		**Baseline**	**1.12 (0.08)**	**0.95, 1.28**		Baseline	1.12 (0.08)	0.95, 1.28	
	Time post-intervention	0.06 (0.13)	-0.19, 0.32	0.630								
	Time follow-up	0.09 (0.14)	-0.37, 0.19	0.534								
	Group×time post-intervention	-0.20 (0.17)	-0.54, 0.15	0.262	**Group×time post-intervention**	**-0.13 (0.13)**	**-0.39, 0.13**	**0.320**	Group×time post intervention & follow-up[‡]	-0.21 (0.12)	-0.43, 0.02	0.067
	Group×time follow-up	-0.22 (0.19)	-0.60, 0.16	0.256	**Group×time follow-up**	**-0.31 (0.14)**	**-0.59, 0.03**	**0.032**				

VO_{2max}[¶]	Baseline	29.74 (1.18)	27.42,32.05		Baseline	29.74 (1.17)	27.45,32.03					
	Time post-intervention	-0.32 (0.70)	-1.69, 1.06	0.652					**Baseline & post-intervention**	**29.91 (1.16)**	**27.64,32.19**	
	Time follow-up	0.54 (0.80)	-1.03, 2.12	0.501								
	Group×time post-intervention	1.22 (1.02)	-0.79, 3.22	0.233	Group×time post-intervention	0.90 (0.76)	-0.59, 2.39	0.235				
	Group×time follow-up	-2.50 (-1.13)	-4.72, -0.27	0.028	Group×time follow-up	-1.96 (0.82)	-3.55,-0.36	0.016	**Group×time follow-up**	**-2.37 (0.74)**	**-3.82, -0.92**	**0.001**

Note. Estimate= Estimated coefficient. SE= Standard error. Baseline was accepted as equal for both groups .Time (baseline=0, post-intervention=1, follow-up=2). Group (AVG=0/ HIIT=1). Group × time post-intervention (time=1, group=HIIT), group × time follow-up (time=2/ group=HIIT)

Non-significant variables were taken out of the model, and the models were compared by using Likelihood ratio test or AIC as suited, until reaching the best fit model for each variable.

Overall symptoms (total PANSS): Model 1 vs model 2, likelihood ratio test p=0.98

[†]Five factor model of PANSS (Wallwork)

Positive factor: Model 1 vs model 2 likelihood ratio test p=0.56

Negative factor: Model 1 vs model 2 likelihood ratio test p= 0.72, model 2 vs model 3 likelihood ratio test p=0.65

Disorganized factor: Model 1 vs model 2 likelihood ratio test p=0.80, model 2 vs model 3 likelihood ratio test p=0.85

Expressed factor: Model 1 vs model 2 likelihood ratio test p=0.38, model 2 vs model 3 likelihood ratio test p=0.15

Depressed factor: Model 1 vs model 2 likelihood ratio test p=0.86, model 2 AIC=940 vs model 3 AIC=938.

[‡] post-intervention and follow up were merged into a single time point (time after baseline) as no changes were detected in either group between these two time points

[§]CDSS: Calgary Depressive Scale for Schizophrenia

[¶] VO2max: Maximum oxygen uptake
